# Growth deficits in cystic fibrosis mice begin *in utero* prior to IGF-1 reduction

**DOI:** 10.1371/journal.pone.0175467

**Published:** 2017-04-06

**Authors:** Rebecca Darrah, Ilya Bederman, Megan Vitko, Dana M. Valerio, Mitchell L. Drumm, Craig A. Hodges

**Affiliations:** 1 Frances Payne Bolton School of Nursing, Case Western Reserve University, Cleveland, Ohio, United States of America; 2 Department of Genetics and Genome Sciences, Case Western Reserve University, Cleveland, Ohio, United States of America; 3 Department of Pediatrics, Case Western Reserve University, Cleveland, Ohio, United States of America; University Medical Center Utrecht, NETHERLANDS

## Abstract

Growth deficits are common in cystic fibrosis (CF), but their cause is complex, with contributions from exocrine pancreatic insufficiency, pulmonary complications, gastrointestinal obstructions, and endocrine abnormalities. The CF mouse model displays similar growth impairment despite exocrine pancreatic function and in the absence of chronic pulmonary infection. The high incidence of intestinal obstruction in the CF mouse has been suggested to significantly contribute to the observed growth deficits. Previous studies by our group have shown that restoration of the cystic fibrosis transmembrane conductance regulator (CFTR) in the intestinal epithelium prevents intestinal obstruction but does not improve growth. In this study, we further investigate growth deficits in CF and gut-corrected CF mice by assessing insulin-like growth factor 1 (IGF-1). IGF-1 levels were significantly decreased in CF and gut-corrected CF adult mice compared to wildtype littermates and were highly correlated with weight. Interestingly, perinatal IGF-1 levels were not significantly different between CF and wildtype littermates, even though growth deficits in CF mice could be detected late in gestation. Since CFTR has been suggested to play a role in water and nutrient exchange in the placenta through its interaction with aquaporins, we analyzed placental aquaporin expression in late-gestation CF and control littermates. While significant differences were observed in *Aquaporin 9* expression in CF placentas in late gestation, there was no evidence of placental fluid exchange differences between CF and control littermates. The results from this study indicate that decreased IGF-1 levels are highly correlated with growth in CF mice, independent of CF intestinal obstruction. However, the perinatal growth deficits that are observed in CF mice are not due to decreased IGF-1 levels or differences in placenta-mediated fluid exchange. Further investigation is necessary to understand the etiology of early growth deficits in CF, as growth has been shown to be a significant factor in disease outcomes.

## Introduction

Cystic fibrosis (CF) is a complex, systemic, and lethal disorder caused by mutations in the cystic fibrosis transmembrane conductance regulator gene (CFTR). Absence of CFTR, a cAMP-regulated anion channel, leads to a plethora of disease manifestations, including growth deficits. The growth deficits in CF patients have been observed as early as birth and include reduced weight, length, and head circumference [1–4]. Reduced growth in CF patients continues throughout life, with approximately 25% of CF children reported below the 10th percentile for weight-for-age and sex, and CF adults typically display reduced body mass index [5]. Improvement in these traits has been observed in recent years due to increased focus on nutrition and improved pancreatic enzyme replacement therapy [6–8]. While CF lung disease is the leading cause of morbidity and mortality in CF, there is a strong correlation between improved growth indices and pulmonary function and overall health [9–13]. These observations indicate that growth deficits in CF patients have significant clinical importance and highlight the necessity of understanding the origins for CF-associated growth reduction.

The growth anomalies of CF are likely multifactorial, a consequence of the many organ systems affected by the disease. More than 85% of CF patients experience exocrine pancreatic insufficiency (PI) due to the destruction of pancreatic acinar cells that occurs early in the disease [14, 15]. The absence of the pancreatic digestive enzymes leads to maldigestion and malabsorption, ultimately leading to malnutrition requiring pancreatic enzyme supplementation. In addition to pancreatic enzyme replacement, dietary augmentation therapy through increased caloric intake is commonly used to increase body mass in CF patients. These clinical measures result in improved weight, but not to non-CF values and do not significantly affect stature, suggesting factors other than malnutrition are involved [7]. Lung disease is also a clear contributor to reduced growth in CF. The lung infection and inflammation in CF result in cachexia that is often associated with chronic illness, diminishing growth and decreasing appetite [16, 17]. However, lung infections do not contribute to neonatal and early childhood growth deficits and CF patients with normal pulmonary function also have reduced growth [18]. While it is clear that PI and lung disease contribute to the overall growth deficit in CF, they alone do not account for the entirety of the reduced growth, which suggests additional contributing factors.

CF animal models can be used to identify and understand these additional factors that affect growth in CF. Reduced growth is common to all CF models, including the mouse, pig, ferret, and rat [19–23]. CF pigs and ferrets display pancreatic and lung disease manifestations similar to CF patients, which can confound the identification of additional factors contributing to reduced growth [20, 21]. CF mice retain exocrine pancreatic function [24–26] and are maintained without chronic lung infection [24, 27–29], the two traits that are most often attributed to growth delay and retardation in humans with CF, yet these animals display severe growth deficits [19, 23, 26, 30]. Therefore, pulmonary and pancreatic disease are likely independent factors that contribute to the reduced growth in CF patients and CF animal models, but do not account for all of the reduced growth in CF.

In this study, we use CF mouse models to further understand the factors that contribute to growth reduction in CF. Our findings indicate that the growth reduction in CF mice correlates with insulin-like growth factor 1 (IGF-1) levels in the juvenile and adult stages. Further, the growth reduction is independent of the incidence of intestinal obstruction that is prevalent in CF models. In addition, our data indicate that CF growth deficits begin late in gestation, prior to reduction of IGF-1 levels, and may be due to the absence of CFTR in the placenta.

## Materials and methods

### Mice

The CF mice (*Cftr*^*invfl10*^) and CF gut-corrected mice (*Cftr*^*invfl10*^+ villin Cre) have been previously described [30]. The *Cftr* allele and Cre transgene were backcrossed to the C57BL/6J background for 10 generations to produce an inbred strain. Animals were monitored on a daily basis, and weight was assessed every 5 days from 10 to 40 days of age. Mice were determined to have succumbed to intestinal obstruction if obvious impaction was observed in the intestine postmortem. Length of 6-wk-old euthanized mice was assessed from nose to anus by use of digital calipers. Weight of inguinal fat weight was also assessed from these 6-wk-old mice. All mouse fetuses and newborn pups were obtained from timed matings of heterozygote males and females and embryonic day 1 was designated as the day after a vaginal plug was identified. Embryonic day 15 (e15) and embryonic day 18 (e18) fetuses were obtained by cesarean section of the euthanized dam and each fetus and placenta was blotted dry and weight was obtained. All mice were genotyped by PCR using a previously described protocol [30]. All animals used in this study were cared for according to a Case Western Reserve University approved protocol and Institutional Animal Care and Use Committee guidelines. Animals were housed in standard polysulfone microisolator cages in ventilated units with corncob bedding. Mice were given *ad libitum* access to chow (Harlan Teklad 7960; Harlan Teklad Global Diets, Madison, WI) and sterile water. All animals were maintained on a 12-h light, 12-h dark schedule at a mean ambient temperature of 22°C.

### Measurement of IGF-1 and Aqps

To evaluate gene expression of *Igf1* and *Aquaporin 1*, *3*, *8* and *9*, RNA was isolated from liver or placenta by use of TRIzol (Invitrogen). One microgram of RNA was reverse transcribed into cDNA by use of QScript cDNA synthesis kit (VWR). Real-time quantitative PCR was performed on a StepOne PCR system (Applied Biosystems). Expression was assessed via TaqMan expression assays for *Igf1*(Mm00439560) or *Aquaporin 1*(Mm01326466), *3*(Mm01208559), *8*(Mm00431846) and *9* (Mm00508097) (Applied Biosystems). Expression was normalized to β-actin as the endogenous reference. Each RNA sample was used to make cDNA in duplicate, and the expression results were then averaged to yield the final result. The average of each sample was then expressed as a percentage of expression from control littermates. Mouse IGF-1 serum levels were determined using a mouse IGF-1 milliplex assay (RMIGF187K; EMD Millipore) measured on a Luminex system. Each sample was run in triplicate and averaged.

### Determination of fetal fluid transfer

To assess fetal fluid transfer from dam to fetus, we employed two methods. First, total water content of placenta and fetus were measured in e18 fetuses. The percent wet weight of each placenta or fetus was determined by dividing the initial weight of each placenta or fetus by the weight of each after desiccation in an oven at 95°C. Second, we used deuterium dilution methodology to determine total body water of each of the e18 fetuses. Pregnant female mice were given an intraperitoneal injection of 800 μl of 99.9% deuterium labeled water (^2^H_2_O). Two hours after injection terminal blood samples were collected from both dam and pups and processed immediately. The ^2^H-labeling of body water was determined by exchange with acetone, as described previously [31]. Briefly, 5 μl of whole blood or standard were reacted for 4 hours at room temperature with 5 μl of 10 N KOH and 5 μl of acetone. The reaction was carried out in a crimped Gas Chromatography-Mass Spectrometry (GC/MS) vial and after 4 hours of incubation, samples were analyzed by GC/MS. All sample analyses were carried out using an Agilent 5973N-MSD mass spectrometer equipped with an Agilent 6890 gas chromatograph with A DB-17MS (Agilent) capillary column (30 m x 0.25 mm x 0.25 mm). Samples were analyzed in Selected Ion Monitoring (SIM) mode using electron impact ionization (EI). Ion dwell time was set to 10 msec. Acetone m/z 58 and 59 were monitored. Isotopic enrichment was determined as ratio of m/z 59/(58+59) and plotted on a standard curve. Data were presented as amount of ^2^H_2_O in the blood of each fetus divided by the amount of ^2^H_2_O in the blood of the corresponding dam (% ^2^H_2_O fetal/dam).

### Statistics

Results are expressed as the mean +/− SEM. Differences between groups were determined using a two-way ANOVA with post-hoc Tukey test. A Spearman Rank Correlation test was utilized for the comparison of IGF1 and weight. A P value of <0.05 was considered significant for these tests. A Bonferroni correction for multiple comparisons was used for assessing significance in *Aqp* expression and a P value of <0.0125 was considered significant.

## Results and discussion

### Reduced growth in CF mice correlates with decreased IGF-1, but is not due to intestinal obstruction

Reduced growth in CF mouse models has been hypothesized to be the result of the high incidence of intestinal obstruction [19, 23, 26, 30]. Using conditional *Cftr* mouse alleles, we previously reported that inactivation of C*ftr* in the intestinal epithelium of mice produced an intestinal obstruction phenotype, but did not reduce growth, while activation of C*ftr* in the intestinal epithelium of mice (gut-corrected CF) resulted in no evidence of intestinal obstruction, but also no improvement in growth [30]. These data using the gut-corrected CF mice were from a mixed mouse strain background (129/sv and C57Bl/6) [30].

**Fig 1:**
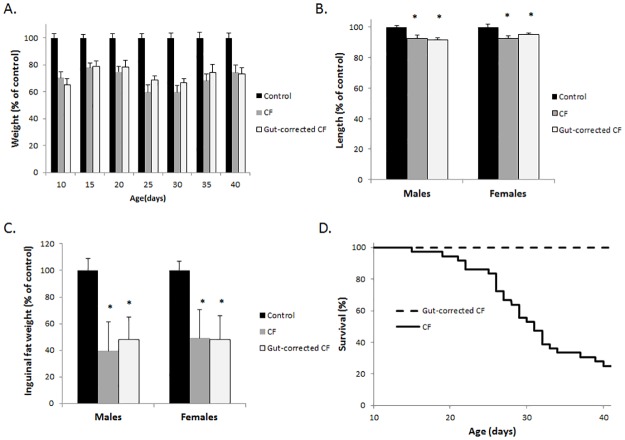
Intestinal correction of CFTR does not improve growth. (A) Weight of CF mice, gut-corrected CF mice and control littermates was assessed up to 40 days of age (n≥10 of each sex). Weight was significantly decreased in CF and gut-corrected CF mice compared to control animals at every age measured (P<0.001) with equal numbers of each sex used. (B) Length and (C) inguinal fat weight of CF mice, gut-corrected CF mice and control littermates was assessed between 6–7 weeks of age (n≥10 of each sex). Both length and inguinal fat weight was significantly decreased in CF and gut-corrected CF mice compared to control animals independent of sex (* = P<0.05; data represent mean±SE). (D) Survival of CF and gut-corrected CF between 10–42 days of age. All deaths were the result of intestinal obstruction as verified by postmortem examination.

To reduce the possibility of genetic heterogeneity as a confounding variable, we backcrossed the conditional Cftr allele and the Cre recombinase transgene necessary to produce the gut-corrected CF mouse to the C57BL/6 background for 10 generations. On the C57BL/6 inbred background, the CF mice and gut-corrected CF mice continued to show very similar growth deficiencies compared to normal littermates (Fig 1). Specifically, the weight of both CF and gut-corrected CF mice was significantly reduced compared to wildtype littermates’ weight measured between 10 and 40 days of age, with both groups in the range of 60–80% of normal mouse weight (Fig 1A). Length was significantly reduced in CF and gut-corrected CF mice independent of sex, with both groups’ length reduced by 5–10% of wildtype littermates’ length. Reduction in fat accumulation is common in CF patients and CF mice and is observed in the gut-corrected CF model as well. Both CF and gut-corrected CF mice display a 50% reduction in inguinal fat (Fig 1C). Although the CF and gut-corrected CF mice were similar in all measured aspects of growth, the incidence of lethal intestinal obstruction differed significantly with 75% of CF affected but none of the gut-corrected CF mice succumbing to obstruction by 6 weeks of age (Fig 1D; P<0.0001 up to 6 weeks of age; n>35 mice per group). In addition, no signs of intestinal obstruction were observed in any gut-corrected CF mice following sacrifice and postmortem examination. These data clearly show that restoration of CFTR in the intestinal epithelium and prevention of intestinal obstruction in CF mice does not improve growth.

This observation is supported by previous studies in which amelioration of the CF intestinal obstruction phenotype occurred through diet alteration, drug treatment, or genetic manipulation. CF mice on a liquid diet [32, 33] or a solid diet with an osmotic laxative in the water [34] have a significant reduction in intestinal obstruction, but still display reduced growth. Similarly, treatment with a novel guluronate oligomer, OligoG, which reduces intestinal mucus accumulation, improved survival in CF mice, but did not restore normal growth [35]. In addition, genetic approaches to reduce intestinal obstruction in CF models have not resulted in a normal growth profile. Overexpression of the mouse *Clca1* (chloride channel accessory 1, previously termed m*Clca3)*, in CF mice decreased intestinal obstruction through an unknown mechanism but did not improve growth at weaning [36]. Likewise, a gut-corrected CF pig model that lacks intestinal obstruction displayed no improvement in growth [37]. In combination with our data, these studies indicate that intestinal obstruction is not the major contributing factor to the reduced growth displayed by CF mice [30]. Other mechanisms, such as the endocrine abnormalities including reduced IGF-1 observed in CF patients and CF animal models, must be investigated as a possible origin for the CF growth defect [34].

As a major effector of growth hormone, IGF-1 plays an integral role in intrauterine, childhood, and pubertal growth [38, 39]. Although structurally and functionally similar to insulin, IGF-1 has a higher growth-promoting activity through cell proliferation, survival, and general growth. IGF-1 synthesis mainly occurs in the liver, but some is also produced in peripheral tissues like cartilage, bone, and some solid organs [40–42]. IGF-1 is known to be reduced in CF patients and CF animal models and has been suggested to be a primary cause for the reduced growth phenotype in CF [34, 43]. However, many factors can lead to a reduction in hormone levels, especially in a disease state, making it difficult to identify cause and effect. For instance, the gastrointestinal disease in CF has been suggested to contribute to the reduced IGF-1 levels [34]. The gut-corrected CF mouse allows for the determination of IGF-1 levels in a CF mouse without gastrointestinal disease.

**Fig 2:**
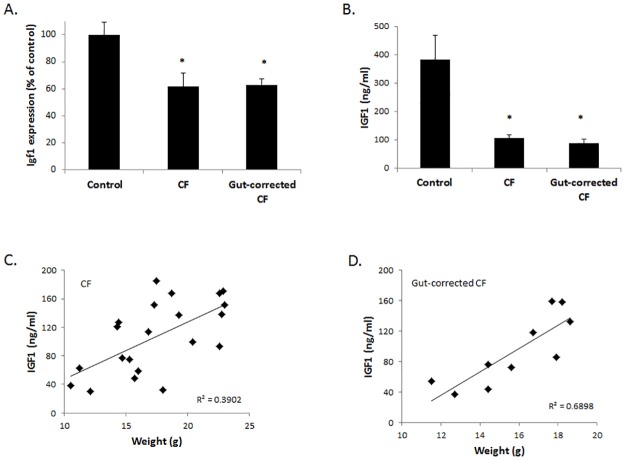
IGF-1 levels are reduced in CF and gut-corrected CF mice and correlate with growth. (A) *Igf1* expression and (B) IGF-1 serum levels are significantly reduced at 6 weeks of age in both CF and gut-corrected CF mice compared to control littermates (* = P<0.05; n≥6; data represent mean±SE). IGF-1 levels were significantly correlated with weight in (C) CF and (D) gut-corrected CF mice (P<0.05; n≥10).

We next evaluated IGF-1 in both CF and gut-corrected CF adult mice and found the levels in liver and blood were significantly decreased in both groups compared to wildtype littermates (40% reduction in liver and 70% reduction in blood; Fig 2A and 2B). In addition, IGF-1 levels did not differ between CF and gut-corrected CF mice. Interestingly, IGF-1 levels were significantly correlated with weight in both groups of CF mice (Fig 2C and 2D). Therefore, both CF and gut-corrected CF mice display similar growth and IGF-1 abnormalities, and while reduced IGF-1 in CF mice correlates with growth, reduced IGF-1 is not due to intestinal disease. While these data indicate a potential causal relationship between reduced IGF-1 and reduced growth in adult CF mice, it is unknown if a similar relationship is evident at birth in CF mice. Understanding the timing of IGF-1 reduction in CF in relationship to growth reduction may provide insight into the underlying mechanism.

### Growth differences in CF mice are evident perinatally, but are not due to IGF-1 differences

**Fig 3:**
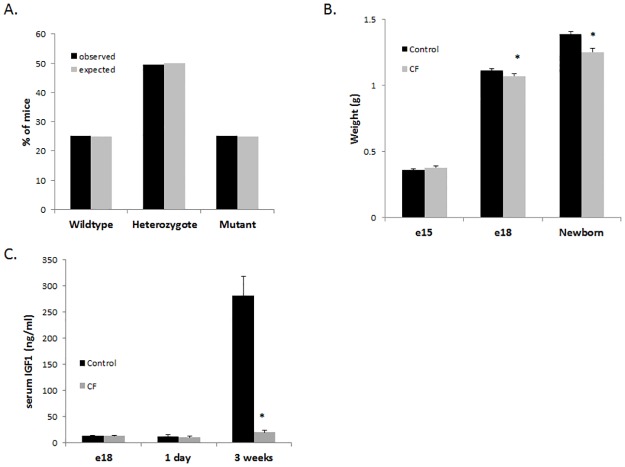
Reduced growth in CF mice occurs prenatally in the absence of reduced IGF-1. (A) A Mendelian distribution was observed in fetuses originating from *Cftr* heterozygote matings. (n>100) (B) Weight was significantly reduced in newborn CF mice and e18 fetuses compared to control littermates but the groups did not differ at e15. (C) IGF-1 levels were not significantly different between CF and control littermates perinatally (e18 and newborn) but IGF-1 levels were significantly reduced between CF and control littermates at 3 weeks of age. (* = P<0.05; n≥15; data represent mean±SE)

In addition to the well-documented growth deficiencies in CF patients throughout postnatal life, reduced birth weight has also been observed, suggesting a prenatal origin for the growth deficiencies [1, 4]. We measured body weight of late-gestation mouse fetuses on embryonic day 15 (e15) and embryonic day 18 (e18) and newborn CF and control pups to determine whether CF mice exhibit the reduced birth weight observed in CF patients, and the timing of this deficit. We observed normal Mendelian distribution of genotypes from late-gestation pups (Fig 3A), indicating that any observed loss of CF pups [26], occurs after birth. Therefore, the data are not biased by potential prenatal loss of CF pups. Reduced weight in CF pups compared to control pups was observed in the e18 fetuses and newborn pups (9% at both), while no differences in weight were observed in the e15 fetuses (Fig 3B). To evaluate whether these weight differences correlated with IGF-1 levels, we measured IGF-1 in the blood from CF and control pups and observed no significant differences in e18 fetuses or newborn pups (Fig 3C). However, IGF-1 levels were significantly reduced in CF mice compared to control mice as early as 3 weeks of age (Fig 3C). Taken together, these data indicate that CF growth deficiency is evident late in gestation and continues through adulthood; however, IGF-1 levels are initially normal prenatally and at birth, but later, reduced. Therefore, IGF-1 reduction is unlikely to be the sole origin for the perinatal CF growth deficiency.

The weight difference between CF and control e18 mouse fetuses, but not between e15 fetuses, demonstrates that reduced growth in CF is not evident until late gestation. This is similar to humans, in which CF newborns have reduced birthweight compared to non-CF newborns, but weight is not significantly different between CF and non-CF preterm infants (gestational age < 37 weeks)[1]. In contrast to the present study, reduced IGF-1 in CF newborns and CF piglets has been noted [43]. Interestingly, unlike CF humans and CF mice, significantly decreased birthweight is not observed in the CF pig [37, 43, 44]. While reduced IGF-1 is a constant among humans, pigs, and mice with CF, the temporal relationship between reduced IGF-1 and reduced growth is complicated, and further study is necessary to elucidate this relationship. Insulin levels, nutrition, and even the presence of meconium ileus (MI), at least in CF humans and pigs, may all play roles in the reduced IGF-1. The absence of MI in CF mice suggests a lack of intestinal pathology *in utero* however intestinal contribution to late gestational growth reduction is possible and cannot be entirely ruled out without further investigation. While there are many possible contributing factors to reduced growth in CF, in the CF mouse model, reduced growth is evident in late gestation and continues into adulthood in the absence of pancreatic, lung, or even intestinal pathology.

### Absence of CFTR in the placenta may contribute to prenatal CF growth differences

**Fig 4:**
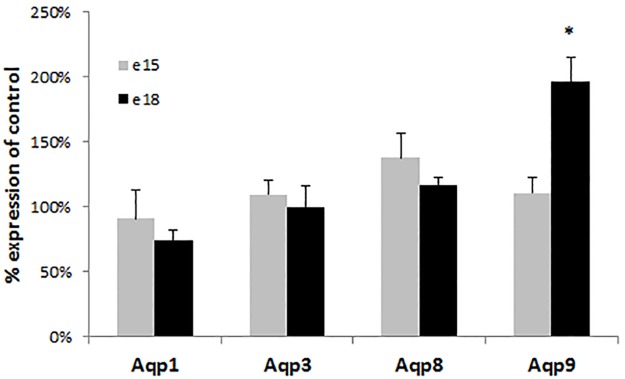
Placental aquaporin expression in CF mice. *Aquaporin(Aqp) 1*,*3*,*8* and *9* expression was evaluated in placentas from e15 and e18 CF and control littermates. Only *Aqp9* at e18 was significantly different between placentas from CF and control littermates (* = P<0.005) Each measurement is an average of six mice completed in replicates (data represent mean±SEM).

The absence of CF disease pathology and normal IGF-1 levels in CF mouse pups suggest other origins for the observed prenatal growth deficits. A role for the placenta in reduced perinatal growth in CF has been postulated [1, 45]. The presence of CFTR in the placenta has been observed, but its exact role in this organ remains unclear [46, 47]. It has been suggested that CFTR plays a role in water and nutrient exchange in the placenta because inhibition of CFTR in placental explants decreases water uptake [48]. Absence of CFTR in the placenta leading to decreased placental mediated fluid exchange may account for the observed late gestation weight deficits in CF mice. In addition, CFTR has been suggested to work in conjunction with specific aquaporins, which facilitate the transfer of water and small solutes to the fetus [48–51]. To evaluate this possibility, we examined the expression of four aquaporins known to be expressed in the placenta in CF and control placentas from e15 and e18 fetuses. While placental aquaporin expression was not different between CF and control littermates at e15, *aquaporin 9* (*Aqp9*) was significantly elevated by almost twofold in the placentas from CF pups at e18 (p<0.0037; Fig 4). This finding is consistent with a previous study which observed that decreased CFTR expression in preeclamptic placentas was associated with increased AQP9 expression despite the loss of AQP9 functionality leading to reduced water transport [48].

**Fig 5:**
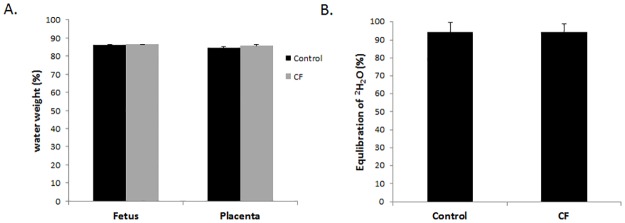
Placental-mediated fluid exchange is not different between CF and control fetuses. (A) Fetal body and placental water weight at e18 was not significantly different between CF and control fetuses. (B) Equilibration of ^2^H_2_O between dam and CF and control fetuses at e18 were not significantly different. (n≥10; data represent mean±SEM).

Given the importance of aquaporins in placental-mediated water exchange for the fetus, we hypothesized that the altered *Aqp9* expression may lead to decreased fluid uptake, leading to the observed weight difference between CF and control fetuses and newborn pups. We calculated fetal fluid transfer using two independent measures: percent water weight and by the isotopic dilution method using deuterium-labeled water (as described in Methods). There were no significant differences between water weight in the body or placenta between the CF and control e18 fetuses (Fig 5A), and there were no detectable differences in fluid exchange between the dam and CF and control littermates (Fig 5B). Therefore, the differences in neonatal birth weight between the CF and control mice are not due to differences in placental water transfer. However, further studies are needed to interpret the increase in *Aqp9* expression in CF placentas given this absence in difference in placental water transfer between CF and control fetuses. For example, it is possible that the increase in *Aqp9* expression does not coincide with an increase in protein levels or, alternatively, increased *Aqp9* expression may lead to increased protein levels with no difference in Aqp9 function. Interestingly, a previous study noted that mothers fed a hypercaloric diet gave birth to CF babies of higher birth weight as compared with mothers on a normal diet, suggesting the possibility of placental nutrient exchange differences in CF still needs to be determined [52].

## Conclusions

The CF and gut-corrected CF mice are indistinguishable in their growth characteristics, indicating, that growth deficits in postnatal CF mice are not due to intestinal complications such as obstruction. In addition, the relationship between decreased IGF-1 and reduced size exists in CF mice, but the growth deficit precedes the reduction in IGF-1 during development. Our data suggest that the late-gestation growth reduction in CF mice, which corresponds to late third trimester in humans, may have a possible placental origin, but the mechanisms remain to be determined.

## References

[1] Festini F, Taccetti G, Repetto T, Reali MF, Campana S, Mergni G, et al Gestational and neonatal characteristics of children with cystic fibrosis: a cohort study. J Pediatr. 2005;147(3):316–20. 10.1016/j.jpeds.2005.04.031 16182668

[2] Ghosal S, Taylor CJ, Pickering M, McGaw J, Beckles-Willson N, Wales JK. Disproportionate head growth retardation in cystic fibrosis. Arch Dis Child. 1995;72(2):150–2. 7702380 10.1136/adc.72.2.150 PMC1511022

[3] Haeusler G, Frisch H, Waldhor T, Gotz M. Perspectives of longitudinal growth in cystic fibrosis from birth to adult age. Eur J Pediatr. 1994;153(3):158–63. 8181496 10.1007/BF01958975

[4] Darrah R, Nelson R, Damato EG, Decker M, Matthews A, Hodges CA. Growth Deficiency in Cystic Fibrosis Is Observable at Birth and Predictive of Early Pulmonary Function. Biol Res Nurs. 2016;18(5):498–504. 10.1177/1099800416643585 27081158 PMC5942479

[5] Cystic Fibrosis Foundation Patient Registry: 2005 Annual Data Report to the Center Directors. Bethesda, MD: Cystic Fibrosis Foundation 2006.

[6] Gaskin KJ. Nutritional care in children with cystic fibrosis: are our patients becoming better? European journal of clinical nutrition. 2013;67(5):558–64. 10.1038/ejcn.2013.20 23462946

[7] Stallings VA, Stark LJ, Robinson KA, Feranchak AP, Quinton H. Evidence-based practice recommendations for nutrition-related management of children and adults with cystic fibrosis and pancreatic insufficiency: results of a systematic review. J Am Diet Assoc. 2008;108(5):832–9. Epub 2008/04/30. 10.1016/j.jada.2008.02.020 18442507

[8] Yen EH, Quinton H, Borowitz D. Better nutritional status in early childhood is associated with improved clinical outcomes and survival in patients with cystic fibrosis. J Pediatr. 2013;162(3):530–5 e1. 10.1016/j.jpeds.2012.08.040 23062247

[9] Corey M, Farewell V. Determinants of mortality from cystic fibrosis in Canada, 1970–1989. Am J Epidemiol. 1996;143(10):1007–17. 8629607 10.1093/oxfordjournals.aje.a008664

[10] Corey M, McLaughlin FJ, Williams M, Levison H. A comparison of survival, growth, and pulmonary function in patients with cystic fibrosis in Boston and Toronto. J Clin Epidemiol. 1988;41(6):583–91. 3260274 10.1016/0895-4356(88)90063-7

[11] Konstan MW, Butler SM, Wohl ME, Stoddard M, Matousek R, Wagener JS, et al Growth and nutritional indexes in early life predict pulmonary function in cystic fibrosis.J Pediatr. 2003;142(6):624–30. 10.1067/mpd.2003.152 12838189

[12] Lai HC, Corey M, FitzSimmons S, Kosorok MR, Farrell PM. Comparison of growth status of patients with cystic fibrosis between the United States and Canada. Am J Clin Nutr. 1999;69(3):531–8. 10075341 10.1093/ajcn/69.3.531

[13] Lai HC, Kosorok MR, Laxova A, Davis LA, FitzSimmon SC, Farrell PM. Nutritional status of patients with cystic fibrosis with meconium ileus: a comparison with patients without meconium ileus and diagnosed early through neonatal screening. Pediatrics. 2000;105(1 Pt 1):53–61. 10617704 10.1542/peds.105.1.53

[14] Durie PR. The pathophysiology of the pancreatic defect in cystic fibrosis. Acta Paediatr Scand Suppl. 1989;363:41–4. 2701923 10.1111/apa.1989.78.s363.41

[15] Kopelman H, Durie P, Gaskin K, Weizman Z, Forstner G. Pancreatic fluid secretion and protein hyperconcentration in cystic fibrosis.N Engl J Med. 1985;312(6):329–34. 10.1056/NEJM198502073120601 3969086

[16] Levy E, Gurbindo C, Lacaille F, Paradis K, Thibault L, Seidman E. Circulating tumor necrosis factor-alpha levels and lipid abnormalities in patients with cystic fibrosis. Pediatr Res. 1993;34(2):162–6. 10.1203/00006450-199308000-00011 8233719

[17] Nixon LS, Yung B, Bell SC, Elborn JS, Shale DJ. Circulating immunoreactive interleukin-6 in cystic fibrosis. Am J Respir Crit Care Med. 1998;157(6 Pt 1):1764–9. 10.1164/ajrccm.157.6.9704086 9620903

[18] Hardin DS, Ferkol T, Ahn C, Dreimane D, Dyson M, Morse M, et al A retrospective study of growth hormone use in adolescents with cystic fibrosis. Clin Endocrinol.2005;62(5):560–6. 10.1111/j.1365-2265.2005.02259.x 15853825

[19] Hodges CA, Cotton CU, Palmert MR, Drumm ML. Generation of a conditional null allele for Cftr in mice. Genesis. 2008;46(10):546–52. 10.1002/dvg.20433 18802965 PMC2711445

[20] Rogers CS, Stoltz DA, Meyerholz DK, Ostedgaard LS, Rokhlina T, Taft PJ, et al Disruption of the CFTR gene produces a model of cystic fibrosis in newborn pigs. Science. 2008;321(5897):1837–41. 10.1126/science.1163600 18818360 PMC2570747

[21] Sun X, Sui H, Fisher JT, Yan Z, Liu X, Cho HJ, et al Disease phenotype of a ferret CFTR-knockout model of cystic fibrosis. J Clin Invest. 2010;120(9):3149–60. 10.1172/JCI43052 20739752 PMC2929732

[22] Tuggle KL, Birket SE, Cui X, Hong J, Warren J, Reid L, et al Characterization of defects in ion transport and tissue development in cystic fibrosis transmembrane conductance regulator (CFTR)-knockout rats. PLoS One. 2014;9(3):e91253 10.1371/journal.pone.0091253 24608905 PMC3946746

[23] Zeiher BG, Eichwald E, Zabner J, Smith JJ, Puga AP, McCray PB Jr., et al A mouse model for the delta F508 allele of cystic fibrosis. J Clin Invest. 1995;96(4):2051–64. 10.1172/JCI118253 7560099 PMC185844

[24] Durie PR, Kent G, Phillips MJ, Ackerley CA. Characteristic multiorgan pathology of cystic fibrosis in a long-living cystic fibrosis transmembrane regulator knockout murine model. Am J Pathol. 2004;164(4):1481–93. Epub 2004/03/25. 10.1016/S0002-9440(10)63234-8 15039235 PMC1615340

[25] Pascua P, Garcia M, Fernandez-Salazar MP, Hernandez-Lorenzo MP, Calvo JJ, Colledge WH, et al Ducts isolated from the pancreas of CFTR-null mice secrete fluid. Pflugers Archiv: European journal of physiology. 2009;459(1):203–14. 10.1007/s00424-009-0704-9 19655163

[26] Snouwaert JN, Brigman KK, Latour AM, Malouf NN, Boucher RC, Smithies O, et al An animal model for cystic fibrosis made by gene targeting. Science. 1992;257(5073):1083–8. 1380723 10.1126/science.257.5073.1083

[27] Cohen JC, Lundblad LK, Bates JH, Levitzky M, Larson JE. The "Goldilocks effect" in cystic fibrosis: identification of a lung phenotype in the cftr knockout and heterozygous mouse. BMC Genet. 2004;5:21 10.1186/1471-2156-5-21 15279681 PMC506778

[28] Darrah RJ, Mitchell AL, Campanaro CK, Barbato ES, Litman P, Sattar A, et al Early pulmonary disease manifestations in cystic fibrosis mice. J Cyst Fibros. 2016. 10.1016/j.jcf.2016.05.002 PMC5121081 27231029

[29] Kent G, Iles R, Bear CE, Huan LJ, Griesenbach U, McKerlie C, et al Lung disease in mice with cystic fibrosis. J Clin Invest. 1997;100(12):3060–9. Epub 1998/01/31. 10.1172/JCI119861 9399953 PMC508519

[30] Hodges CA, Grady BR, Mishra K, Cotton CU, Drumm ML. Cystic fibrosis growth retardation is not correlated with loss of Cftr in the intestinal epithelium. Am J Physiol Gastrointest Liver Physiol. 2011;301(3):G528–36. Epub 2011/06/11. 10.1152/ajpgi.00052.2011 21659619 PMC3174541

[31] Shah V, Herath K, Previs SF, Hubbard BK, Roddy TP. Headspace analyses of acetone: a rapid method for measuring the 2H-labeling of water. Anal Biochem. 2010;404(2):235–7. 10.1016/j.ab.2010.05.010 20488158

[32] Eckman EA, Cotton CU, Kube DM, Davis PB. Dietary changes improve survival of CFTR S489X homozygous mutant mouse. Am J Physiol. 1995;269(5 Pt 1):L625–30. 7491981 10.1152/ajplung.1995.269.5.L625

[33] van Heeckeren AM, Schluchter MD, Drumm ML, Davis PB. Role of Cftr genotype in the response to chronic Pseudomonas aeruginosa lung infection in mice. Am J Physiol Lung Cell Mol Physiol. 2004;287(5):L944–52. Epub 2004/07/13. 10.1152/ajplung.00387.2003 15246977

[34] Rosenberg LA, Schluchter MD, Parlow AF, Drumm ML. Mouse as a model of growth retardation in cystic fibrosis. Pediatr Res. 2006;59(2):191–5. Epub 2006/01/28. 10.1203/01.pdr.0000196720.25938.be 16439577

[35] Vitko M, Valerio DM, Rye PD, Onsoyen E, Myrset AH, Dessen A, et al A novel guluronate oligomer improves intestinal transit and survival in cystic fibrosis mice.J Cyst Fibros. 2016. 10.1016/j.jcf.2016.06.005 27343003

[36] Young FD, Newbigging S, Choi C, Keet M, Kent G, Rozmahel RF. Amelioration of cystic fibrosis intestinal mucous disease in mice by restoration of mCLCA3. Gastroenterology. 2007;133(6):1928–37. 10.1053/j.gastro.2007.10.007 18054564

[37] Stoltz DA, Rokhlina T, Ernst SE, Pezzulo AA, Ostedgaard LS, Karp PH, et al Intestinal CFTR expression alleviates meconium ileus in cystic fibrosis pigs. J Clin Invest. 2013;123(6):2685–93. 10.1172/JCI68867 23676501 PMC3668832

[38] Agrogiannis GD, Sifakis S, Patsouris ES, Konstantinidou AE. Insulin-like growth factors in embryonic and fetal growth and skeletal development (Review). Mol Med Rep. 2014;10(2):579–84. 10.3892/mmr.2014.2258 24859417 PMC4094767

[39] Netchine I, Azzi S, Le Bouc Y, Savage MO. IGF1 molecular anomalies demonstrate its critical role in fetal, postnatal growth and brain development. Best Pract Res Clin Endocrinol Metab. 2011;25(1):181–90. 10.1016/j.beem.2010.08.005 21396584

[40] Han VK, Lund PK, Lee DC, D'Ercole AJ. Expression of somatomedin/insulin-like growth factor messenger ribonucleic acids in the human fetus: identification, characterization, and tissue distribution. J Clin Endocrinolo Metab. 1988;66(2):422–9. 10.1210/jcem-66-2-422 2448331

[41] Roberts CT Jr., Lasky SR, Lowe WL Jr., Seaman WT, LeRoith D. Molecular cloning of rat insulin-like growth factor I complementary deoxyribonucleic acids: differential messenger ribonucleic acid processing and regulation by growth hormone in extrahepatic tissues. Mol Endocrinol. 1987;1(3):243–8. 10.1210/mend-1-3-243 3453891

[42] Yakar S, Liu JL, Stannard B, Butler A, Accili D, Sauer B, et al Normal growth and development in the absence of hepatic insulin-like growth factor I. Proc Natl Acad Sci U S A. 1999;96(13):7324–9. 10377413 10.1073/pnas.96.13.7324 PMC22084

[43] Rogan MP, Reznikov LR, Pezzulo AA, Gansemer ND, Samuel M, Prather RS, et al Pigs and humans with cystic fibrosis have reduced insulin-like growth factor 1 (IGF1) levels at birth. Proc Natl Acad Sci U S A. 2010. Epub 2010/11/10. 10.1073/pnas.1015281107 PMC2996661 21059918

[44] Rogers CS, Hao Y, Rokhlina T, Samuel M, Stoltz DA, Li Y, et al Production of CFTR-null and CFTR-DeltaF508 heterozygous pigs by adeno-associated virus-mediated gene targeting and somatic cell nuclear transfer. J Clin Invest. 2008;118(4):1571–7. 10.1172/JCI34773 18324337 PMC2265103

[45] Davis B, Shennan DB, Boyd CA. Chloride transport in cystic fibrosis placenta. Lancet. 1985;1(8425):392–3. 10.1016/s0140-6736(85)91407-2 2857438

[46] Faller DP, Egan DA, Ryan MP. Evidence for location of the CFTR in human placental apical membrane vesicles. Am J Physiol. 1995;269(1 Pt 1):C148–55. 7543241 10.1152/ajpcell.1995.269.1.C148

[47] Mylona P, Glazier JD, Greenwood SL, Sides MK, Sibley CP. Expression of the cystic fibrosis (CF) and multidrug resistance (MDR1) genes during development and differentiation in the human placenta. Mol Hum Reprod. 1996;2(9):693–8. 9239684 10.1093/molehr/2.9.693

[48] Castro-Parodi M, Levi L, Dietrich V, Zotta E, Damiano AE. CFTR may modulate AQP9 functionality in preeclamptic placentas. Placenta. 2009;30(7):642–8. 10.1016/j.placenta.2009.04.012 19481256

[49] Cheung KH, Leung CT, Leung GP, Wong PY. Synergistic effects of cystic fibrosis transmembrane conductance regulator and aquaporin-9 in the rat epididymis. Biol Reprod. 2003;68(5):1505–10. 10.1095/biolreprod.102.010017 12606488

[50] Pietrement C, Da Silva N, Silberstein C, James M, Marsolais M, Van Hoek A, et al Role of NHERF1, cystic fibrosis transmembrane conductance regulator, and cAMP in the regulation of aquaporin 9. J Biol Chem. 2008;283(5):2986–96. 10.1074/jbc.M704678200 18055461

[51] Schreiber R, Nitschke R, Greger R, Kunzelmann K. The cystic fibrosis transmembrane conductance regulator activates aquaporin 3 in airway epithelial cells. J Biol Chem. 1999;274(17):11811–6. 10206998 10.1074/jbc.274.17.11811

[52] Boyer PH. Low birth weight in fibrocystic disease of the pancreas. Pediatrics. 1955;16(6):778–84. 13273117

